# Differential expression of osteopontin, and osteoprotegerin mRNA in epicardial adipose tissue between patients with severe coronary artery disease and aortic valvular stenosis: association with HDL subclasses

**DOI:** 10.1186/s12944-017-0550-2

**Published:** 2017-08-18

**Authors:** María Luna-Luna, David Cruz-Robles, Nydia Ávila-Vanzzini, Valentín Herrera-Alarcón, Jesús Martínez-Reding, Sergio Criales-Vera, Julio Sandoval-Zárate, Jesús Vargas-Barrón, Carlos Martínez-Sánchez, Armando Roberto Tovar-Palacio, José Manuel Fragoso, Elizabeth Carreón-Torres, Gilberto Vargas-Alarcón, Óscar Pérez-Méndez

**Affiliations:** 1 Department of Molecular Biology, Instituto Nacional de Cardiología “Ignacio Chávez”, Juan Badiano 1, Sección XVI, 14080 México City, D.F. Mexico; 2 Department of Echocardiography, Instituto Nacional de Cardiología “Ignacio Chávez”, México City, Mexico; 3 Department of Cardiothoracic Surgery, Instituto Nacional de Cardiología “Ignacio Chávez”, México City, Mexico; 4 Department of Adult Cardiology, Instituto Nacional de Cardiología “Ignacio Chávez”, México City, Mexico; 5 Department of Radiology, Instituto Nacional de Cardiología “Ignacio Chávez”, México City, Mexico; 6 Department of Cardiopulmonary, Instituto Nacional de Cardiología “Ignacio Chávez”, México City, Mexico; 7 Department of Emergency, Instituto Nacional de Cardiología “Ignacio Chávez”, México City, Mexico; 8 Department of Physiology of Nutrition, Instituto Nacional de Ciencias Médicas y Nutrición “Salvador Zubirán”, México City, Mexico; 9 Study Group of Atherosclerosis, Instituto Nacional de Cardiología “Ignacio Chávez”, México City, Mexico

**Keywords:** Adipose tissue, High-density lipoprotein, Osteopontin, Osteonectin, Osteoprotegerin

## Abstract

**Background:**

Previous studies suggest a relationship of the epicardial adipose tissue (EAT) with progression and calcification of the atherosclerotic plaque; however, it is unknown if this tissue expresses genes that may participate on these processes and if the expression of these genes is regulated by high-density lipoprotein (HDL) subclasses.

**Methods:**

To explore this possibility, we determined the mRNA expression by qPCR of a pro-calcifying gene (osteopontin (*OPN*)), and two anti-calcifying genes (osteoprotegerin (*OPG*) and osteonectin (*ON*)), in biopsies of EAT obtained from 15 patients with coronary artery disease (CAD) determined by angiography, and 15 patients with diagnostic of aortic valve stenosis but without CAD as control group. We determined the distribution and composition of HDL subclasses by electrophoresis and their statistical relationship with the gene expression in EAT.

**Results:**

EAT from CAD patients showed a higher expression level of *OPN* and *OPG* than control group, whereas *ON* expression was similar between groups. Large HDL subclasses were cholesterol-poor in CAD patients as estimated by the cholesterol-to-phospholipid ratio. A linear regression model showed an independent association of *OPN* expression with HDL3a-cholesterol, and *OPG* expression with the relative proportion of HDL3b protein. Logistic analysis determined that *OPN* expression was positively associated with the presence of atherosclerotic plaque

**Conclusion:**

*OPN, ON,* and *OPG* genes are transcribed in EAT; to the exception of *ON*, the level of expression was different in CAD patients and control group, and correlated with some HDL subclasses, suggesting a new role of these lipoproteins.

**Electronic supplementary material:**

The online version of this article (doi:10.1186/s12944-017-0550-2) contains supplementary material, which is available to authorized users.

## Background

Epicardial adipose tissue (EAT) is found in the atrio- and inter-ventricular grooves, and closely surrounds the coronary arteries, even invading the adventitia in some sections [1]. Atherosclerotic lesions are present in sections of the artery adjacent to EAT [1–4], whereas these lesions are never found in regions of the artery that sink into the myocardium. These evidences suggest that EAT contributes to the development and calcification of the atherosclerotic plaque, probably interacting with the coronary arteries through a paracrine signaling [2]. Thereby, EAT may be a source of expression of genes that encodes pro- and anti-calcifying proteins such as osteopontin, osteoprotegerin, and osteonectin.

Osteopontin is encoded by *OPN* gene, and it is a multi-functional protein [5] that promotes the inflammatory micro-environment characteristic of the atherosclerosis [6]. In addition, several studies have demonstrated that osteopontin increases with progression and calcification of the atherosclerotic plaque [6–8], suggesting a pro-calcifying role of this protein.

Osteoprotegerin is encoded by *OPG* gene and it is member of the family of TNF receptor [9]. Osteoprotegerin has been considered an anti-calcifying protein since it prevents the receptor activator of NF-κB (RANK) binds to its ligand (RANKL) [10]. By this mechanism, osteoprotegerin inhibits the transcription of pro-calcifying genes as *bone morphogenetic protein- 4 (BMP-4)* and *alkaline phosphatase (ALPL)* [11, 12]. In addition, osteoprotegerin may interact with TNF-related apoptosis inducing ligand (TRAIL) and avoid the formation of anti-apoptotic bodies, which can be mineralized [13–15].

*ON* gene encodes for osteonectin that has been considered as an anti-calcifying protein since the level of expression of *ON* decreases with the progression of atherosclerosis and it is lower in calcified atherosclerotic plaques [8].

Several tissues express *OPN*, *OPG*, and *ON* [10, 16–19] but little is known about the expression of these genes in EAT; in this context, the secretion of osteoprotegerin is induced by high-density lipoprotein (HDL) in in vitro cultures [20]. These data suggest that HDLs protect against the progression of atheroma calcification through mechanisms that evoke the regulation of genes related with this process. HDLs constitute a heterogeneous family of lipoproteins that may be classified by size in HDL2b, HDL2a, HDL3a, HDL3b and HDL3c [21, 22]. These subclasses differ in their lipid and protein content, and they may have different anti-atherogenic characteristics [23–25]. In agreement with this idea, a previous report from our group demonstrated that asymptomatic subjects with high coronary calcium score had lower number of HDL particles and a high proportion of small HDL subclasses enriched with triglycerides [26]; HDL subclasses may be indirect players in the calcification process of the atherosclerotic plaque through the regulation of the expression of genes that encode pro- and anti-calcifying proteins.

Therefore, the aim of this study was to determine whether the mRNA expression levels of *OPN*, *OPG* and *ON* in EAT from patients with coronary artery disease (CAD) is different from that of subjects without coronary lesions determined by angiography. We further determined the potential statistical association of mRNA levels in EAT and HDL subclasses.

## Methods

### Patients

We obtained EAT biopsies from 30 patients, 15 with coronary artery disease (CAD) candidate to revascularization surgery. As comparison (named control) group, we enrolled 15 patients with diagnostic of aortic valve stenosis, without CAD and who were programmed for chirurgical valve replacement; since the etiology of aortic valve stenosis is similar to that of the CAD, this comparison group is of particular relevance to focus on the potential paracrine contribution of EAT to the atherosclerotic lesion.

The presence and complexity of CAD was determined through angiography and SYNTAX score, respectively. Anthropometric parameters included body mass index (BMI) and blood pressure. The exclusion criteria were patients with thyroid, renal, or hepatic disease, and patients with previous coronary angioplasty or previous revascularization surgery. The estimated glomerular filtration rate (eGFR) was calculated according to CKD-EPI method [27].

Epicardial adipose tissue biopsies were obtained from the atrio- interventricular groove during surgery and placed immediately in tubes with RNA*later*™ Stabilization Solution (Life technologies, Carlsbad, CA, USA) and frozen at −20 °C for further analysis.

This study was approved by the Ethics Committee of the National Institute of Cardiology “Ignacio Chávez” with the number 13–818. Participants were informed about the objectives of the study and those who agreed to participate signed a letter of informed consent.

### Laboratory assessment

Blood samples were obtained in EDTA tubes after 12 h overnight fasting, centrifuged to 4 °C, the plasma was separated and analyzed. Plasma total cholesterol (TC), triglycerides (TG) and glucose were determined by commercially available enzymatic colorimetric methods (Randox LTD, Crumlin, UK). For lipids associated to HDLs, the phosphotungstic acid-Mg^2+^ method was used to precipitate apo B-containing lipoproteins before quantifying cholesterol, triglycerides (TG) (Randox, UK), phospholipids (PH) (Wako Chemicals, Richmond, VA) and free cholesterol (FC) (using non-commercially available enzymatic mixtures recently developed by our group) plasma concentrations. Esterified HDL-cholesterol was calculated as the difference between total cholesterol and free cholesterol concentrations multiplied by a factor of 1.68 to be expressed in milligrams per deciliter (mg/dL). Low-density lipoprotein-cholesterol (LDL-C) was estimated with the formula of Friedewald.

### Quantification of mRNA

Total RNA was isolated from tissue samples using the RiboPure™ RNA Purification Kit following the manufacturer’s instructions (Ambion, Life Technologies, Carlsbad, CA, USA). RNA quantity and purity was assessed spectrophotometrically using Nanodrop technology. RNA was reverse transcribed using SuperScript® VILO™ cDNA Synthesis Kit (Invitrogen, Life Technologies, Carlsbad, CA, USA) in line with the manufacturer’s protocol. qPCR of *OPN*, *ON* and *OPG* mRNA was performed using TaqMan Gene Expression Assays (Invitrogen/Life Technologies, Carlsbad, CA) and compared with the housekeeping gene *GAPDH*. The primers used were Hs00959010_m1 (*OPN*)**;** Hs00234160_m1 (*ON*); Hs00900358_m1 (*OPG*); and Hs0275899_g1 (*GAPDH*) all specific for human. Quantification of gene expression was calculated by the standard curve method and normalized to *GAPDH*.

### Isolation of HDL particles and analysis of their structure

HDL were separated by ultracentrifugation and dialyzed against 0.09 M Tris/0.08 M Boric acid/3 mM EDTA buffer, pH 8.4 [28]. The homogeneity and hydrodynamic diameter of HDL subclasses were estimated as previously described [28]. Briefly, HDL samples were separated according to their size by non-denaturing 3% – 30% polyacrylamide gradient gel electrophoresis. Gels were stained for total cholesterol, free cholesterol, phospholipids, and triglycerides using methods recently developed by our group [28]. For the classification of the HDL subclasses, we considered the following size intervals: HDL3c, 7.94–8.45 nm; HDL3b, 8.45–8.98 nm; HDL3a, 8.98–9.94 nm; HDL2a, 9.94–10.58 nm; and HDL2b, 10.58–13.59 nm [28].

### Statistical analysis

Normally distributed data, determined with a Kolmogorov–Smirnov test, were expressed as means and standard error (SE). To determine if the contribution of the drug consumption, smoking habit, and the presence of diabetes were statistically significant, we performed a χ^2^ test. Student’s unpaired t-test was used for comparison between groups. Pearson’s correlation coefficients were determined for the expression levels and dimensional variables. A stepwise linear multiple regression model was used to examine the impact of variables on mRNA expression levels. The variables selected for the multiple regression analyses were those with a significant correlation in the bivariate analysis including potential confounding variables to correct the model. The relationship between the presence of atherosclerotic plaque and gene expression was explored using binary logistic regression; the results derived from this analysis were expressed as odds ratio and 95% confidence intervals. All reported *P*-values were based on two-sided tests. *P* < 0.05 values were considered as significant. Analyses were performed with the Statistical Package for the Social Sciences version 21.0 (SPSS, Inc., Chicago, IL, USA).

## Results

Patient’s anthropometric and biochemical data are shown in Table 1. Mean age and systolic blood pressure were comparable between CAD and control groups, whereas body mass index (BMI) was significantly higher in CAD patients. Drugs intake and smoking habit were similar in both groups (Table 1). Only two individuals in control group reported excessive alcohol intake occasionally. On the basis of the hormone plasma levels and estimated GFR, thyroid and renal function were normal in both groups (Table 1). On the other hand, CAD group had lower plasma concentrations of total cholesterol (30%) and LDL-C (36%), and higher concentrations of glucose than control group.

**Table 1:** Clinical characteristics

	Controls (*n* = 15)	CAD (*n* = 15)
Age (years)	58.86 ± 2.19	61.58 ± 2.60
Sex (M/F)	11/4	10/5
BMI (kg/m^2^)	25.69 ± 1.01	29.66 ± 1.28*
Statin use, *n* (%)	3 (20%)	3 (20%)
Anti-hypertensive agents, *n* (%)	10 (67%)	9 (60%)
Anti-platelet agents, *n* (%)	2 (13%)	4 (27%)
Anti- diabetic agents, *n* (%)	4 (27%)	6 (40%)
Organic Nitrates, *n* (%)	2 (13%)	3 (20%)
Aspirin, *n* (%)	5 (33%)	6 (40%)
DM2, *n* (%)	6 (40%)	8 (53%)
Active smokers, *n* (%)	3 (20%)	2 (13%)
SBP (mmHg)	122.93 ± 4.26	127.50 ± 5.38
DBP (mmHg)	73.93 ± 3.79	75.33 ± 3.99
Creatinine (mg/dL)	0.86 ± 0.06	0.87 ± 0.07
eGFR (mL/min/1.73 m2)	60.00 ± 0.01	59.80 ± 0.20
TSH (mIU/L)	2.42 ± 0.52	3.33 ± 0.45
T4 (μg/dL)	8.12 ± 0.86	8.12 ± 0.45
T3 (ng/mL)	1.16 ± 0.04	1.14 ± 0.02
Cholesterol (mg/dL)	147.82 ± 13.28	103.08 ± 14.73*
Triglycerides (mg/dL)	99.09 ± 6.49	100.40 ± 6.49
Glucose (mg/dL)	92.84 ± 2.55	106.21 ± 6.19*
HDL-cholesterol (mg/dL)	40.67 ± 3.20	31.32 ± 2.17*
Free HDL-cholesterol (mg/dL)	10.36 ± 1.71	11.77 ± 1.04
Esterified HDL-cholesterol (mg/dL)	50.91 ± 5.37	32.84 ± 4.54*
HDL- triglycerides (mg/dL)	29.63 ± 3.13	24.78 ± 1.99
HDL-phospholipids (mg/dL)	70.31 ± 5.04	70.41 ± 5.33
LDL-cholesterol (mg/dL)	98.18 ± 6.65	62.51 ± 4.70*
HDL 2b (%)	17.59 ± 0.82	20.83 ± 1.90
HDL 2a (%)	11.40 ± 0.30	12.25 ± 0.46
HDL 3a (%)	26.82 ± 1.03	27.05 ± 0.40
HDL 3b (%)	18.69 ± 0.40	20.29 ± 0.61*
HDL 3c (%)	24.49 ± 1.31	20.22 ± 1.44*
SYNTAX score	0.00 (0.00–0.00)	10.00 (2.00–44.00)

*CAD* coronary artery disease; *BMI* body mass index; *DM2* type 2 diabetes mellitus; *SBP* systolic blood pressure; *HDL* high density lipoprotein; *LDL* low density lipoprotein; *M* male; *F *female; *eGFR* estimated glomerular filtration rate; *TSH* thyroid stimulating hormone; *T4* thyroxine; *T3* triiodothyronine

Data are shown as mean ± SE. Student’s t test *p < 0.05 vs. control group

The lipid profile of the HDLs showed lower plasma concentrations of total cholesterol and esterified cholesterol in the CAD patients compared with the control group (Table 1). These alterations in the lipid profile suggested modifications of HDL subclasses; therefore, we analyzed the lipid composition of these subclasses, and results are shown in the Table 2. The plasma concentrations of total cholesterol and esterified cholesterol of HDL2a, HDL3a, and small HDL3b and 3c were lower in CAD patients than in control group. The concentration of triglycerides was lower in the HDL3b and 3c subclasses (28 and 62%, respectively) in the CAD group whilst the phospholipids and free cholesterol were comparable between both groups (Table 2).

**Table 2:** Lipid plasma concentrations of HDL subclasses

	Group	Subclasses				
		HDL 2b	HDL 2a	HDL 3a	HDL 3b	HDL 3c
TC (mg/dL)	Control	11.74 ± 1.11	5.71 ± 0.44	11.19 ± 0.80	6.16 ± 0.47	6.16 ± 0.42
	CAD	9.27 ± 1.12	4.26 ± 0.25*	7.92 ± 0.64*	4.59 ± 0.35*	3.43 ± 0.24†
FC (mg/dL)	Control	3.53 ± 0.55	1.34 ± 0.19	2.64 ± 0.45	1.08 ± 0.18	1.13 ± 0.16
	CAD	4.00 ± 0.34	1.60 ± 0.13	3.06 ± 0.27	1.46 ± 0.12	1.14 ± 0.11
EC (mg/dL)	Control	14.62 ± 1.84	7.33 ± 0.72	13.73 ± 1.53	8.66 ± 0.73	8.02 ± 0.57
	CAD	8.26 ± 1.85	4.47 ± 0.43*	9.55 ± 1.01*	5.29 ± 0.59*	3.84 ± 0.33†
TG (mg/dL)	Control	8.56 ± 1.10	4.63 ± 0.56	8.40 ± 0.79	4.13 ± 0.46	4.37 ± 0.45
	CAD	10.14 ± 0.97	4.20 ± 0.43	6.56 ± 0.43	2.97 ± 0.21*	1.66 ± 0.23†
PH (mg/dL)	Control	21.08 ± 1.07	9.52 ± 0.54	18.20 ± 1.12	10.11 ± 0.90	10.65 ± 1.52
	CAD	25.55 ± 2.00	10.42 ± 0.76	16.58 ± 1.23	8.86 ± 0.66	7.18 ± 0.63

*TC* total cholesterol; *FC* free cholesterol; *EC* esterified cholesterol; *TG* triglycerides; *PH* phospholipids

Data are shown as mean ± SE. *n* = 15 by group. Student’s t test †*p* < 0.01, and *p < 0.05 vs. control group

We further calculated the cholesterol-to-phospholipids (TC-to-PH) and triglycerides-to-phospholipids (TG-to-PH) ratios as an approach of the HDLs composition (Table 3). The TC-to-PH ratio from CAD group was significantly lower in the HDL2b, 2a and 3a while the TG-to-PH ratio of the HDL3c was lower compared with the control group. The relative size distribution of HDL subclasses, expressed as percentage of the total HDL protein, was only slightly different between groups; CAD group had a higher proportion of the HDL3b (9%) concomitant with a lower proportion of HDL3c (17%) than control group (Table 1).

**Table 3:** Cholesterol-to-phospholipids (TC-to-PH) and triglycerides-to-phospholipids (TG-to-PH) plasma concentration ratios of the different HDL subclasses

Ratio	Group	Subclasses				
		HDL 2b	HDL 2a	HDL 3a	HDL 3b	HDL 3c
TC/PH	Control	0.55 ± 0.04	0.60 ± 0.04	0.59 ± 0.03	0.57 ± 0.02	0.57 ± 0.06
	CAD	0.38 ± 0.05*	0.43 ± 0.03*	0.49 ± 0.03*	0.53 ± 0.03	0.48 ± 0.05
TG/PH	Control	0.36 ± 0.05	0.42 ± 0.07	0.46 ± 0.05	0.42 ± 0.05	0.41 ± 0.06
	CAD	0.41 ± 0.04	0.38 ± 0.03	0.41 ± 0.03	0.35 ± 0.03	0.24 ± 0.03*

Data are shown as mean ± SE. *n* = 15 by group. Student’s t test *p < 0.05 vs. control group

**Fig. 1:**
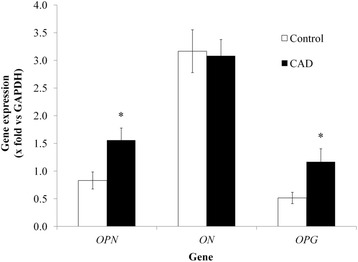
Gene expression for *OPN*, *ON* and *OPG*. Data represent mean ± SE. *n* = 15 by group. Student’s t test (*p < 0.05). *OPN*: *osteopontin, ON: osteonectin, OPG: osteoprotegerin*

The relative mRNA abundance in adipose tissue is shown in Fig. 1. *OPN* expression was 80% higher in the epicardial adipose tissue from CAD group compared with the control subjects. *OPG* was expressed twice in the CAD group than in the EAT obtained from the control group (Fig. 1), whereas *ON* expression was not different between groups.

To explore whether there was a statistical relationship between HDL subclasses and gene expression, we performed a correlation analysis; both, the proportion and lipid composition of the HDL subclasses were associated with the expression of these genes (see Additional file 1). Notably, the relative proportion of the HDL3a and 3b subclasses correlated with *ON* expression (*r* = 0.643, *p* = 0.001; *r* = 0.484, *p* = 0.016, respectively) while HDL3c had a negative correlation with *OPG* expression (*r* = −0.430, *p* = 0.032). Plasma concentrations of total cholesterol associated with HDL3a and 3b correlated negatively with *OPN* expression (*r* = −0.567, *p* = 0.004; *r* = −0.454, *p* = 0.026, respectively).

To identify the potential independent factors that determine the gene expression, we performed a linear multiple regression analysis, considering BMI as covariable; the expression level of each gene was included as dependent variable and the parameters that correlated significantly in the bivariate analysis as independent variables (Additional file 1: Table S1). Lipids associated to total HDLs and those associated to HDL subclasses were included in two different models because of the co-linearity between these parameters. We did not find any association between plasma total HDL-lipids and gene expression (data not shown). Relative size proportion of the HDL3a was a predictor of the *ON* expression whilst HDL3b was for *OPG* expression (Table 4). We performed a second linear regression analysis including the cholesterol associated to HDL subpopulations in order to determine which of the HDL subclasses were specifically associated with the gene expression; only HDL3a-TC was associated with *OPN* expression (Table 4).

**Table 4:** Linear regression model to evaluate the independent parameters associated with the gene expression

Gene	Parameter	B	β	r ^2^	*p*- value	CI
*OPN*	HDL3a-TC	−0.141	−0.587	0.315	0.003	[−0.227 − −0.055]
*ON*	HDL 3a	0.266	0.602	0.334	0.002	[0.110–0.421]
*OPG*	HDL 3b	0.227	0.580	0.313	0.003	[0.086–0.367]

*OPN osteopontin*; *ON osteonectin*; *OPG osteoprotegerin*; *TC* total cholesterol; *HDL* high-density lipoprotein; *CI* confidence interval

We further analyzed whether the gene expression was related with the presence of significant coronary lesions. In the logistic regression analysis, we included the presence of significant coronary lesions as dependent variable and the mRNA expression of the analyzed genes as independent variables. This analysis demonstrated that only *OPN* expression was positively associated with the presence of atherosclerotic plaque (Odds ratio = 5.625, 95% CI [1.077–29.371], *p* = 0.041).

## Discussion

In this study, we demonstrated that the epicardial adipose tissue (EAT) expressed the mRNA of genes related with calcification of the atheroma as *OPN*, *ON* and *OPG*, and that the presence of atherosclerotic plaques was associated with the gene expression of *OPN*. We also demonstrated that the composition and relative size distribution of the HDL correlated with the expression of the genes analyzed, suggesting an additional function of these lipoproteins.

Patients with aortic valve stenosis without significant coronary lesions were included as non-CAD group. The reasons to include this kind of patients were: 1) the possibility to obtain the corresponding biopsy because they underwent a cardiac surgical intervention, and 2) this disease shares many clinical risk factors and features with atherosclerosis such as lipid infiltration and inflammation [29, 30], whereas the topology of lesion (i.e. juxtaposition to epicardial adipose tissue) was the main difference between CAD patients and patients with valvulopathy; in the aortic patients, the calcified lesions are far from EAT. Moreover, patients with aortic valve stenosis lacked of significant coronary lesions as determined by the SYNTAX score = 0 in all these patients at the time of the study. These characteristics made patients with aortic valve stenosis as a suitable group to contrast the findings in CAD patients for the purposes of this study.

The topology of atherosclerosis and the presence of some bone proteins in the subendothelium suggests that the EAT could act as a paracrine tissue for its anatomical contiguity with coronary arteries [2]. To our knowledge, there is not a precedent study that determines the expression of genes encoding for pro- and anti-calcifying proteins in epicardial adipose tissue. Our results demonstrated higher expression levels of *OPN* mRNA in epicardial adipose tissue from CAD group compared to the control group. In addition to its pro-inflammatory role [5, 31], phosphorylated osteopontin is able to inhibit the formation of hydroxyapatite crystals, whereas unphosphorylated isoforms act as pro-calcifying proteins [32, 33]. Taking into account the higher expression of *OPN* mRNA in CAD patients, we speculate that osteopontin present in the atheroma is not phosphorylated. In addition, three splicing variants of the human *OPN* transcript have been identified [34], and the role of such isoforms in the atherosclerosis has not been elucidated; thereby, it is necessary to determine the degree of phosphorylation and isoforms of osteopontin secreted by EAT in further studies.

Our results also demonstrated that epicardial adipose tissue expressed *OPG*; studies in *OPG-ApoE-KO* mice evidenced that calcified area was greater than in *ApoE-KO* mice [35]. Osteoprotegerin is considered an anti-calcifying protein that restrains the differentiation of the vascular smooth muscle cells (VSMC) towards an osteogenic phenotype via of the inhibition of pro-calcifying gene *BMP-4* [12]. Paradoxically, our results demonstrated that *OPG* expression levels were higher in CAD patients than in controls; it should be considered that other proteins such as osteopontin induce the expression of *OPG* [36]. Accordingly, higher *OPG* expression levels in the EAT from CAD patients could be a mechanism to counterbalance the effects pro-inflammatory and pro-calcifying of osteopontin. More studies in vitro and in vivo are needed to evaluate this possibility.

Our results also showed that the gene expression of *ON* mRNA was expressed at similar levels in both, CAD and control group. The role of osteonectin on the calcification process is controversial [8, 26, 37, 38] and may be determined by the presence of other related proteins; more studies are required to establish whether or not the osteonectin has any contribution in the etiology of the atheroma.

A previous study from our group demonstrated a negative correlation between calcium score and HDL2a-, 3a- y 3b–phospholipids [26]. In addition, subjects with high-coronary artery calcium score (CAC) had a lower number of HDL particles than subjects with CAC = 0 [26]. These results suggested that HDL particles regulate the expression of genes encoding for proteins that participate in the formation of the atheroma and its calcification. In order to gain more insight about this possibility, we performed the determination of the composition and size distribution of HDL in both groups of the present study. CAD patients showed lower HDL-cholesterol plasma concentrations than the control group. For a long time, it has been considered to HDL-cholesterol as marker of CAD risk [39, 40]; however, the contribution of the HDL to retard the atherosclerotic process is still controversial. Therefore, it is necessary to determine all lipid components to identify the characteristics that better denote their anti-atherogenic role. In this way, CAD patients had lower concentrations of HDL-EC without significant changes in TG and PH. These differences in plasma concentrations indicate HDL subclasses alterations. For this reason, we further determined HDL subclasses lipid composition.

Esterified cholesterol of the intermediate size HDLs was lower in the CAD group than in controls whereas free cholesterol did not contribute to the changes in plasma levels. In addition, triglycerides plasma concentrations of small HDL particles were lower in the CAD group than in control group, supporting the idea of a significant change of HDLs structure in CAD patients. Therefore, we calculated the TC-to-PH and TG-to-PH ratios; plasma concentrations of phospholipids of HDL subclasses are essential to maintain the structure and size of these lipoproteins; thus, phospholipids of HDL subclasses have been considered as a marker of the number of particles in plasma [41]. In this context, four HDL subclasses were poor in cholesterol while HDL3c had a low content of triglycerides. The observed lipid modifications were accompanied by a slight displacement of HDL subclasses towards large HDLs. Since HDLs structure seems to be associated with the anti-atherosclerotic role of these lipoproteins [41], it is likely that the differences between HDL subclasses in CAD and controls are related with the development of the atheroma in coronary arteries.

Recently, it has been demonstrated that HDL induce the secretion of osteoprotegerin in cultured aortic valve myofibroblasts [20]; the correlations between the calcium score and phospholipids of HDL subclasses previously reported [26] are in line with those evidences. Our data demonstrated a positive correlation between the gene expression of *OPG* and the relative proportion of the HDL3b as well as with HDL-FC of the small particles. Such results further support the possible contribution of these lipoproteins to the calcification process. Moreover, the multivariate analysis showed that for each 1 mg/dL of HDL3a- cholesterol plasma concentration increases, *OPN* expression decreased 0.137 units. Furthermore, every 1% of increment in the proportion of HDL3a and 3b resulted in an increment of 0.273 and 0.222 times of the *ON* and *OPG* expression, respectively. These statistical associations suggest a new role of the HDLs in the progression of atherosclerosis through of the regulation of the expression of pro- and anti-calcifying genes in the EAT.

We recognize as an important limitation of the present study that mRNA levels are not always proportional to the amount of the secreted protein; this is only an initial approach to the possible contribution of EAT to the development of CAD. The lack of the coronary calcium score is also an important limitation of our study as well as the limited number of patients. Finally, it is necessary to determine if the EAT volume is associated with expression of the genes analyzed.

## Conclusion

In conclusion, the EAT expresses the mRNA of three genes that have been related with the calcification process, and that such expression is statistically associated with some components of HDL subclasses. These results suggest a contribution of the EAT to the calcification of the atherosclerotic plaque and a new role of the HDLs.

## Additional file

Additional file 1: Table S1. Bivariate correlation analysis between gene expression and subclasses of HDLs. Only statistically significant correlations are shown. TG: triglycerides, TC: total cholesterol, FC: free cholesterol, EC: esterified cholesterol, HDL: high-density lipoprotein (DOCX 17 kb)

## Abbreviations

CAC: coronary artery calcium
CAD: coronary artery disease
EAT: epicardial adipose tissue
EC: esterified cholesterol
FC: free cholesterol
*GAPDH*: glyceraldehyde-3-phosphate dehydrogenase gene
*ON*: osteonectin gene
*OPG*: osteoprotegerin gene
*OPN*: osteopontin gene
PH: phospholipids
RANKL: the receptor activator of NF-κB ligand
TC: total cholesterol
TRAIL: TNF-alfa related apoptosis-inducing ligand
VSMC: vascular smooth muscle cells
