# Supplementary material for: Differential expression of osteopontin, and osteoprotegerin mRNA in epicardial adipose tissue between patients with severe coronary artery disease and aortic valvular stenosis: association with HDL subclasses
Source: Lipids Health Dis. 2017 Aug 18;16:156. doi: 10.1186/s12944-017-0550-2 (PMC5563041; doi:10.1186/s12944-017-0550-2)
Supplement: Additional file 1: Table S1. — Bivariate correlation analysis between gene expression and subclasses of HDLs. Only statistically significant correlations are shown. TG: triglycerides, TC: total cholesterol, FC: free cholesterol, EC: esterified cholesterol, HDL: high-density lipoprotein (DOCX 17 kb) [file 12944_2017_550_MOESM1_ESM.docx]

Table S1 (available on line).- Bivariate correlation analysis between gene expression and subclasses of HDLs

| Parameter | Gene | | | |
| --- | --- | --- | --- | --- |
|  | *OPN* | *ON* | *OPG* | *TNF-α* |
| TG |  | r=0.468  p=0.021 |  |  |
| G | r= 0.475  p=0.019 |  | r= 0.750  p=0.000 |  |
| TC-HDL | r=-0.549  p=0.005 |  |  |  |
| FC-HDL |  |  |  | r= -0.569  p= 0.002 |
| EC-HDL | r= -0.601  p= 0.002 |  |  |  |
| HDL 3a |  | r= 0.643  p=0.001 |  |  |
| HDL 3b |  | r= 0.484  p=0.016 | r= 0.583  p=0.002 |  |
| HDL 3c |  |  | r= -0.430  p=0.032 |  |
| TC-HDL 2a | r= -0.591  p=0.002 |  |  |  |
| TC-HDL 3a | r= -0.567  p= 0.004 |  |  |  |
| TC-HDL 3b | r= -0.454  p= 0.026 |  |  |  |
| FC-HDL 2b |  |  |  | r= -0.666  p=0.000 |
| FC-HDL 2a |  |  |  | r= -0.552  p=0.003 |
| FC-HDL 3a |  |  | r= 0.398  p=0.049 | r= -0.570  p= 0.002 |
| FC-HDL 3b |  |  | r= 0.420  p=0.041 | r= -0,480  p=0.015 |
| FC-HDL 3c |  |  |  | r= -0.535  p= 0.005 |
| TG-HDL 2a |  |  |  | r= 0.513  p= 0.007 |
| TG-HDL 3c |  |  |  | r= 0.465  p= 0.017 |
| EC-HDL 2b |  |  |  | r= 0.483  p= 0.012 |
| EC-HDL 2a | r= -0.600  p= 0.002 |  |  | r= 0.394  p=0.046 |
| EC-HDL 3b | r= -0.542  p= 0.006 |  |  |  |
| EC-HDL 3c |  |  |  | r= 0.459  p= 0.018 |

TG: triglycerides, G:glucose, TC: total cholesterol, FC: free cholesterol, EC: esterified cholesterol, HDL: high density lipoprotein
